# Sarcomatous Transformation of Recurrent Scapular Osteochondroma in a Patient with the Hereditary Multiple Osteochondromas: A Case Report and Literature Review

**DOI:** 10.7759/cureus.6308

**Published:** 2019-12-06

**Authors:** Sadia Sajid, Amman Yousaf, Usman Nabi, Amir Shahbaz, Umar Amin

**Affiliations:** 1 Radiology, Hamad Medical Corporation, Doha, QAT; 2 Radiology, Hamad General Hospital, Doha, QAT; 3 Internal Medicine, Allama Iqbal Medical College, Lahore, PAK; 4 Radiology, Quaid-e-Azam International Hospital, Islamabad, PAK

**Keywords:** osteochondroma, chondrosarcoma, hereditory multiple exostoses, role of diagnostic imaging, tumor suppressor genes, secondary osseous malignancy, prognostic factors, surgical excision, autosomal dominant, scapular tumor

## Abstract

Hereditary multiple osteochondromas (HMO) is an autosomal dominant disease diagnosed by the presence of two or more than two osteochondromas on radiographs. The majority of cases are asymptomatic. The presence of bony growth, pain, and compression of the surrounding structure are the usual presentations. Malignant transformation into chondrosarcoma is the most feared complication. A rapid increase in size, recurrence after the surgical excision, and infiltrating mass may suggest the conversion into chondrosarcoma. Radiological imaging helps in diagnosing malignant transformation. MRI is the investigation of choice to exclude cancer. We hereby present a case of multiple osteochondromas with suspected malignant transformation due to rapidly increasing painful osseous swelling.

## Introduction

Osteochondromas are benign cartilage capped osseous tumors representing 10% of all bone tumors [1, 2]. Osteochondromas usually present during the first two decades and are comparatively more prevalent in males, with male to female ratio 1.5:1 [1]. Hereditary multiple exostoses (HME) is an autosomal dominant heterogeneous disorder and is diagnosed radiographically by the presence of two or more than two osteochondromas and is associated with mutation of tumor suppressor genes [3]. Osteochondromas mostly involve long bones and are usually asymptomatic but can present with pain due to compression and irritation of the nearby neurovascular structures, a fracture through the stalk of the tumor or bursitis due to chronic friction, and functional impairment [4]. The transformation of an osteochondroma into secondary chondrosarcoma is a rare but grave complication [3]. Detailed physical examination, in addition to radiological imaging modalities, can be helpful in early diagnosis in suspected cases, especially in patients with multiple rapidly growing lesions and family history of malignant transformation [4]. At-risk patients kept under surveillance for early diagnosis of chondrosarcoma [5]. We presented a case of chondrosarcoma of scapula secondary to hereditary multiple osteochondromas.

## Case presentation

**Figure 1:**
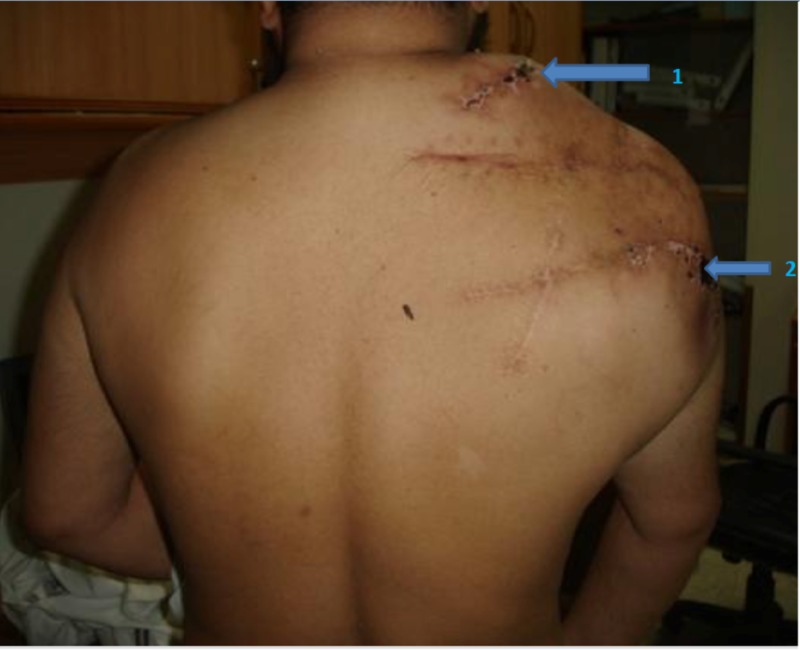
Patient's posterior view Multiple scar marks of previous surgeries (arrow 2) with swelling and deformity of the right suprascapular region (arrow 1).

A 43-year-old Asian male patient with known multiple osteochondromas since childhood presented to the orthopedic clinic with increasing pain and recurrent swelling in the right shoulder. He underwent several surgeries of the right scapula and shoulder for recurrent bony growths. He had many family members with the same pathology. On physical examination, the patient had multiple scar marks of previous surgeries on the shoulder and posterior scapular region with painful swelling and deformity of the right shoulder (Figure 1).

**Figure 2:**
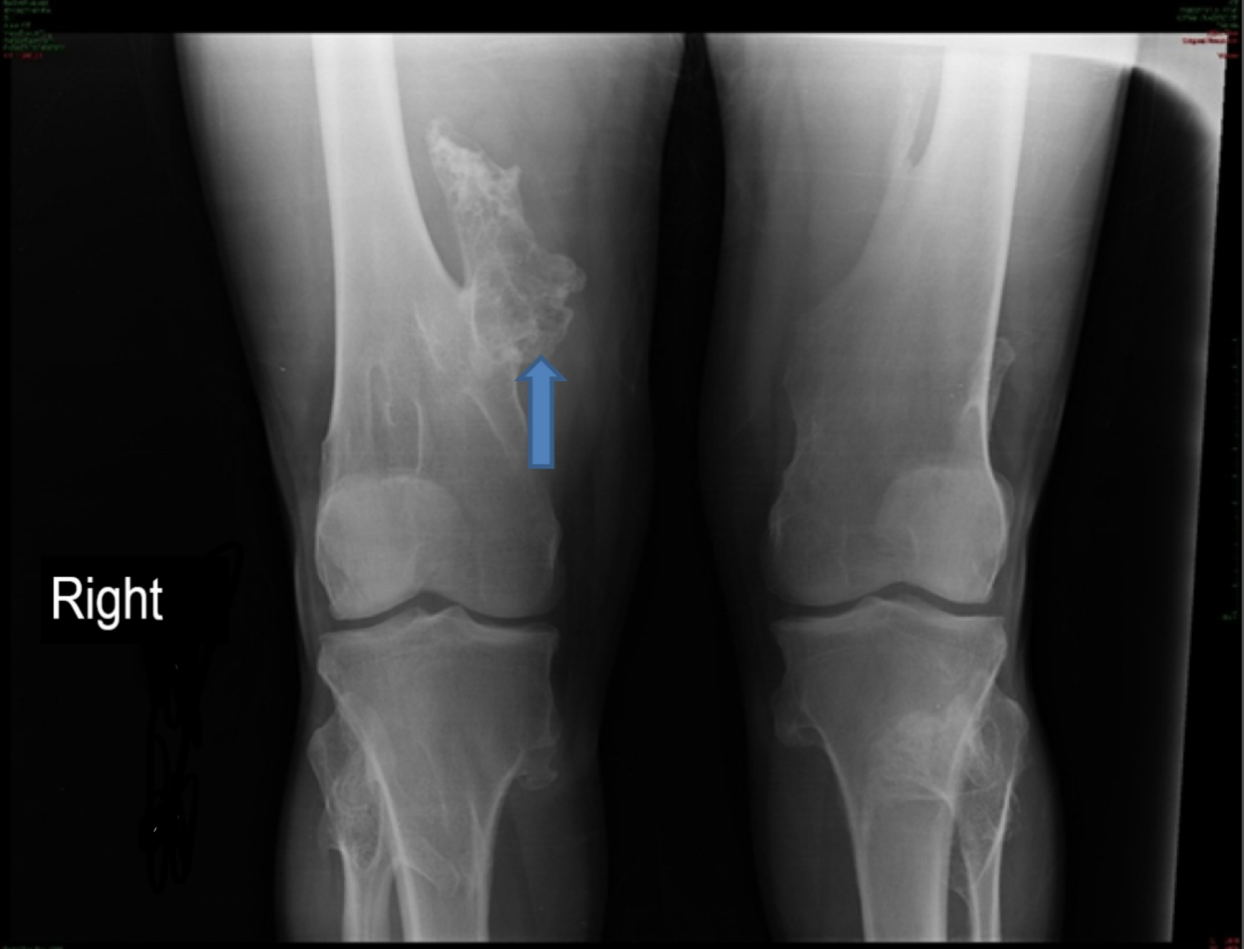
X-ray of both knee joints Multiple exostoses involving bilateral distal femur, proximal tibia, and fibula, the largest is arising from the right distal femur (blue arrow).

The earlier X-ray of the right lower limb showed multiple osteochondromas involving femur, tibia, and fibula of both legs; the larger one was at the distal end of the right femur (Figure 2).

**Figure 3:**
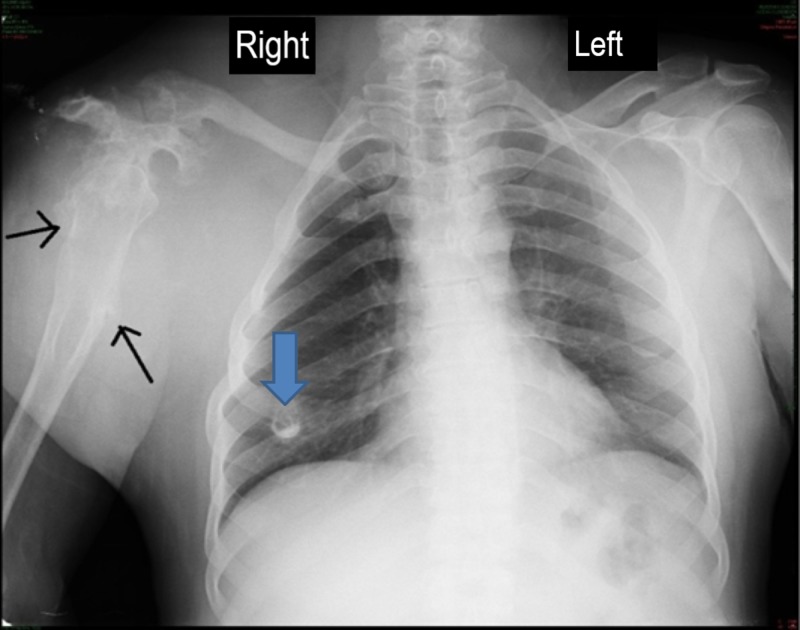
Chest X-ray (Posteroanterior view) Osteochondromas are seen involving right-sided rib (blue arrow) and right humerus (black arrows).

A chest X-ray with exposure of the proximal humerus bones showed destruction and deformity of the right shoulder joint and proximal humerus with marked soft tissue swelling (Figure 3).

**Figure 4:**
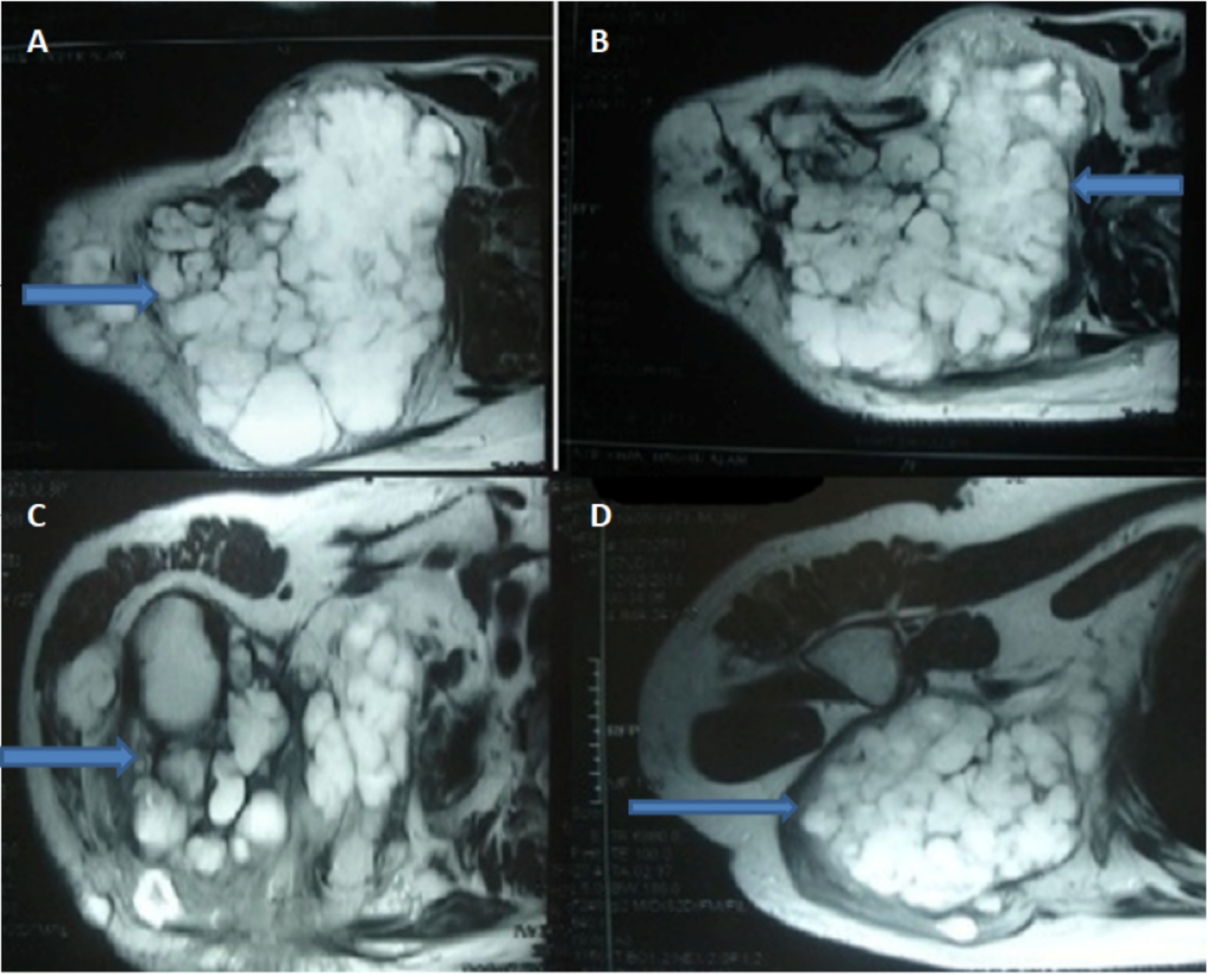
MRI of the right shoulder T2 weighted axial images of right shoulder showing a lobulated hyperintense mass lesion that is totally replacing the right scapula and extending into the suprascapular region and invading subcutaneous soft tissues of the right chest wall, pleura, and lungs (A and B). The mass is extending to the superior aspect of the right shoulder (C), and right axillary region (D).

The patient history of recurrent swelling and chest X-ray findings raised the suspicion of malignant transformation of osteochondroma into chondrosarcoma. Lately, he developed a nodularity in the neck and underwent MRI of the right shoulder to rule out the malignant transformation (Figure 4).

Because of the extensive infiltrative nature and location of the mass, surgery was considered not to be the best initial treatment. The patient underwent three cycles of radiotherapy. However, at six months follow-up, the response was not satisfactory. The patient case reviewed in the multidisciplinary meeting. Different treatment options were discussed with the patient including surgical excision. The patient refused any further workup or treatment and chose conservative management with oral analgesics.

## Discussion

Hereditary multiple exostoses (HME), also known as hereditary multiple osteochondromas (HMO), diaphyseal aclasis, osteochondromatosis, and multiple cartilaginous exostoses, is an autosomal dominant disease of bones. HME lesions are metaphyseal and oriented away from the joint [2, 6]. About 50% of the HME cases diagnosed before five years of age, and more than 80% diagnosed by the end of the first decade [7]. Mutation in tumor suppressor genes exostosin gen 1 (on chromosome 8) and exostosin gen 2 (on chromosome 11) thought to play a pivot role in pathogenesis [7, 8]. Around 30% of mutations are spontaneous, while the rest of 70% have a familial autosomal dominant inheritance. Due to incomplete penetrance, and the presence or absence of EXT- 1 and EXT- 2 clinical manifestation varies. The majority of the cases are asymptomatic. Presentation is more severe among males, especially if they develop at an older age or whether the EXT- 1 is present [7]. The malignant transformation occurs in 4 to 5% cases of HME, while it is less than 1% in solitary osteochondromas [2-3, 9]. About 90% of the secondary malignancy is chondrosarcoma, while rest are due to osteosarcoma, spindle cell sarcoma, and fibrosarcoma [2, 10].

The most common locations of secondary osteochondroma are pelvic bones and proximal femur [3]. The involvement of scapula in secondary chondrosarcoma is relatively rare [11]. Although there are no specific clinical criteria that precisely indicate the presence of secondary chondrosarcoma, a rapid increase in the size of the tumor, recurrence after resection, painful mass, and neurologic signs and symptoms suggest malignant transformation [12]. The underlying mechanism of malignant transformation in HME is still unclear.

Mutation of EXT- 1 and EXT- 2 tumor suppressor genes thought to be associated with secondary chondrosarcoma, but recent studies show that malignant transformation can occur independently of these mutations [12]. The thickness of the cartilage greater than 1.5 cm can be associated with greater malignant potential. However, data regarding the predictability of malignancy is controversial. Therefore, patients with multiple osteochondromas should receive yearly surveillance by detailed clinical examination and radiological imaging. Early detection results in better prognosis and less aggressive surgical intervention [1, 3, 13]. X-ray, CT, and MR can all be used for surveillance and also for the diagnosis of the osteochondromas. The X-ray and CT might show ring and arc or popcorn-like calcifications. CT gives better visualization and typically shows cortical breach. On MR imaging, cartilaginous matrix manifests appear as high T2 and low T1 signal areas, representing the high water content of the hyaline cartilage, and calcifications appear as hypointense on all pulse sequences. The presence of early and exponential enhancement is significantly related to a malignant cartilage-forming tumor. Pathologically chondrosarcoma is multilobulated with peripheral endochondral ossification and central high water content. These findings account for the high T2 MRI signal and popcorn calcification [2, 14-15].

The data on the incidence of chondrosarcoma of the scapula is limited. Scapular chondrosarcoma has a better prognosis as compared to pelvic chondrosarcoma because of the superficial location of the bone [16]. It is less likely to infiltrate the vital structures as ribs prevent its infiltration into the chest cavity. The closest structures that it can infiltrate are axillary vessels and brachial plexus that mostly happens in high-grade tumors [16]. The imaging modalities utilized for the early detection of osteosarcoma. Surgical resection with wide tumor-free margins gives better outcomes. Chondrosarcomas are relatively radiotherapy resistant, and data on the efficacy of chemotherapy is also limited [13].

## Conclusions

Hereditary multiple exostoses (HME) is an autosomal dominant disorder of bones. An increase in the size of bony growth and recurrence after the surgery suggests a malignant transformation. MRI and histopathology should be offered to see the local infiltration of the surrounding vital structures and for grading, as recurrent chondrosarcomas are of high grade and have a poor prognosis.
